# Hospital-Onset Bacteremia at Stony Brook University Hospital

**DOI:** 10.1017/ash.2025.398

**Published:** 2025-09-24

**Authors:** Samantha Rennekamp, Arthur Totten, Tamasin Adams

**Affiliations:** 1 Stony Brook University Hospital

## Abstract

**Introduction:** A vital factor indicating quality of care in hospital settings is hospital-acquired infections (HAI) occurrence, particularly bloodstream infections (BSI). Currently, BSI tracking through the National Healthcare Safety Network (NHSN) focuses primarily on central-line-associated BSI (CLABSI) and MRSA BSIs. Hospital-onset bacteremia (HOB) is a more comprehensive measure of HAI-BSI from all sources. Non-reportable BSIs account for a substantial number of HAIs and contribute significantly to patient outcomes, making them an important component for quality measurement and patient care improvement. NHSN has indicated that HOB reporting will be implemented within the next few years. **Methods:** This study establishes a baseline measure of HOB at Stony Brook University Hospital (SBUH) for 2022 and 2023. HOB cases were defined as any inpatient having at least one positive blood culture result, with the first positive culture collected ≥ 3 days after admission. Patient demographics, length of stay (LOS), and ICU admission status were compared among HOB and community-onset BSI (CO BSI) cases. Case mix index (CMI)-adjusted rates of HOB infection were generated for each hospital location by residualizing the rate of HOB infection on average annual CMI for each unit. Bivariate analyses were used to examine which hospital locations and medical devices were most frequently associated with HOB and CO BSI cases. Causative organisms were also examined. **Results:** A total of 1906 inpatients had positive blood cultures in 2022, 319 (16.74%) were HOB. In 2023, 1853 inpatients had positive cultures, 268 (14.46%) were HOB. Patients with HOB were significantly younger, and Medicare recipients represented the highest proportion of HOB cases. In both years, over 60% of HOB cases were admitted to an ICU compared to about 30% of CO BSI cases, LOS was about 3 times longer, and ICU LOS was more than two times greater for HOB cases compared to CO BSI cases. CMI-adjusted HOB infection rates were highest for MICU, SICU, CICU, and Oncology units, as well as one general medicine unit. All 9 medical devices examined were significantly associated with HOB in bivariate analysis, with central and peripheral IV catheters, urinary devices, arterial lines, and enteral tubes being most frequently present. Probable contaminant organisms were detected in > 50% of all positive cultures examined, but only probable pathogens were detected in > 50% of HOB cases. **Conclusions:** HOB has a significant impact on SBUH inpatients. Results from our study should be used to target infection prevention initiatives moving forward.

